# Artificial intelligence in critical illness and its impact on patient care: a comprehensive review

**DOI:** 10.3389/fmed.2023.1176192

**Published:** 2023-04-20

**Authors:** Muhammad Saqib, Muhammad Iftikhar, Fnu Neha, Fnu Karishma, Hassan Mumtaz

**Affiliations:** ^1^Khyber Medical College, Peshawar, Khyber Pakhtunkhwa, Pakistan; ^2^Ghulam Muhammad Mahar Medical College, Sukkur, Sindh, Pakistan; ^3^Jinnah Sindh Medical University, Karachi, Sindh, Pakistan; ^4^Health Services Academy, Islamabad, Pakistan

**Keywords:** artificial intelligence, intensive care units, critical illness, risk assessment, decision making

## Abstract

Artificial intelligence (AI) has great potential to improve the field of critical care and enhance patient outcomes. This paper provides an overview of current and future applications of AI in critical illness and its impact on patient care, including its use in perceiving disease, predicting changes in pathological processes, and assisting in clinical decision-making. To achieve this, it is important to ensure that the reasoning behind AI-generated recommendations is comprehensible and transparent and that AI systems are designed to be reliable and robust in the care of critically ill patients. These challenges must be addressed through research and the development of quality control measures to ensure that AI is used in a safe and effective manner. In conclusion, this paper highlights the numerous opportunities and potential applications of AI in critical care and provides guidance for future research and development in this field. By enabling the perception of disease, predicting changes in pathological processes, and assisting in the resolution of clinical decisions, AI has the potential to revolutionize patient care for critically ill patients and improve the efficiency of health systems.

## 1. Introduction

The word Artificial Intelligence (AI) describes the methods by which a system may imitate human cognitive functions, such as reasoning capacity, decision-making, generalization, or learning from past experiences, to achieve goals without being expressly programmed for specific activities. AI is characterized as intelligent machines, as opposed to the intelligence of individuals or other living things (1). The areas of learning algorithms, processing natural languages, and robotics may thus fall under the umbrella of artificial intelligence (AI), which has the potential to advance biomedical research, primary care, and health systems. These fields can be adapted to almost any area of medicine.

One of the most hotly contested uses of artificial intelligence (AI) in the healthcare industry has been the development of technology. The use of software, algorithms for machine learning, or artificial intelligence (AI) to simulate mental abilities in the interpretation, evaluation, and comprehension of healthcare data is referred to as AI in healthcare. For instance, AI-based medical algorithms used in mammograms help radiologists by providing a second opinion while aiding in the diagnosis of breast cancer (1).

AI was used in the healthcare industry to produce well-performing medicine. For instance, Insilico Medical has created AI algorithms that can halt viral infection. By providing nutritional guidance to expectant mothers based on their health state and algorithm estimates, another proposal seeks to safeguard them. Epileptic seizure detection, another excellent use of AI, assisted in lessening the severity of epileptic convulsions. With AI and the creation of a cutting-edge movement-detecting device, early stroke might also be accurately predicted.

Although using AI in medical healthcare seems to have the potential to drastically increase the effectiveness of clinical diagnosis and biomedicine in general, it has also raised some ethical questions. One of the main obstacles for medical AI is safety. IBM Watson for oncology is a very good example. It uses AI algorithms to analyse data from patient records and assist physicians in exploring cancer options for treatment for their patient populations. However, it has since come under fire for allegedly making risky and unreliable cancer therapy recommendations.

The quality of medical treatment for critically ill patients has greatly improved due to advancements in care standards (1). Despite this progress, traditional critical care has limitations in fully understanding and addressing the complexities of patients’ health, predicting deterioration, and providing timely treatment. The advent of advanced monitoring systems and non-invasive and invasive treatments has improved bedside care, but it is yet to be determined if these advancements represent the next step in critical care medicine. Artificial intelligence (AI) aims to help computers identify patterns in complex and diverse data, which was once only possible in limited fields like physics or astronomy due to limited computing resources. However, with the recent growth in computing power, AI can now be applied to other fields, including critical care medicine, where there is an abundance of complex data (2). According to a recent study (3), the number of articles about AI in the field of critical care medicine (CCM) has been increasing rapidly, particularly from 2018 to 2020. The majority of these articles are of high quality and come from top-ranked journals. Research into artificial intelligence (AI) has shown promise in terms of predicting disease outcomes and improving patient care (3).

While there are increasing numbers of studies using AI-powered models in the intensive care unit (ICU), our understanding of AI’s potential in critical care is still limited. Additionally, there are challenges that AI must overcome before becoming a routine part of clinical practice. Using the most recent literature, this review aims to improve understanding of the applications of AI in critical illness and its impact on patient care, and it makes recommendations for the future.

## 2. Methods

A comprehensive search was carried out in PubMed, Google Scholar, PLOS One, and Scopus for all relevant literature using the following terms: “critical care,” “intensive care medicine,” “ICU medicine,” “artificial intelligence,” “AI,” “machine learning,” and “critical illness” from January 2018 through February 2023 in the English language. Similar articles were also reviewed using the suggested articles for each paper, and gray literature was also searched using relevant terms. All papers were imported into reference management software, and duplicates were removed. Older versions of the same papers were not included if newer versions were available. All relevant papers were read, and corresponding authors were contacted using email if the full text of a paper was not available. No unpublished papers were included in our review.

## 3. Applications of AI in critical care patient management

**Figure 1:**
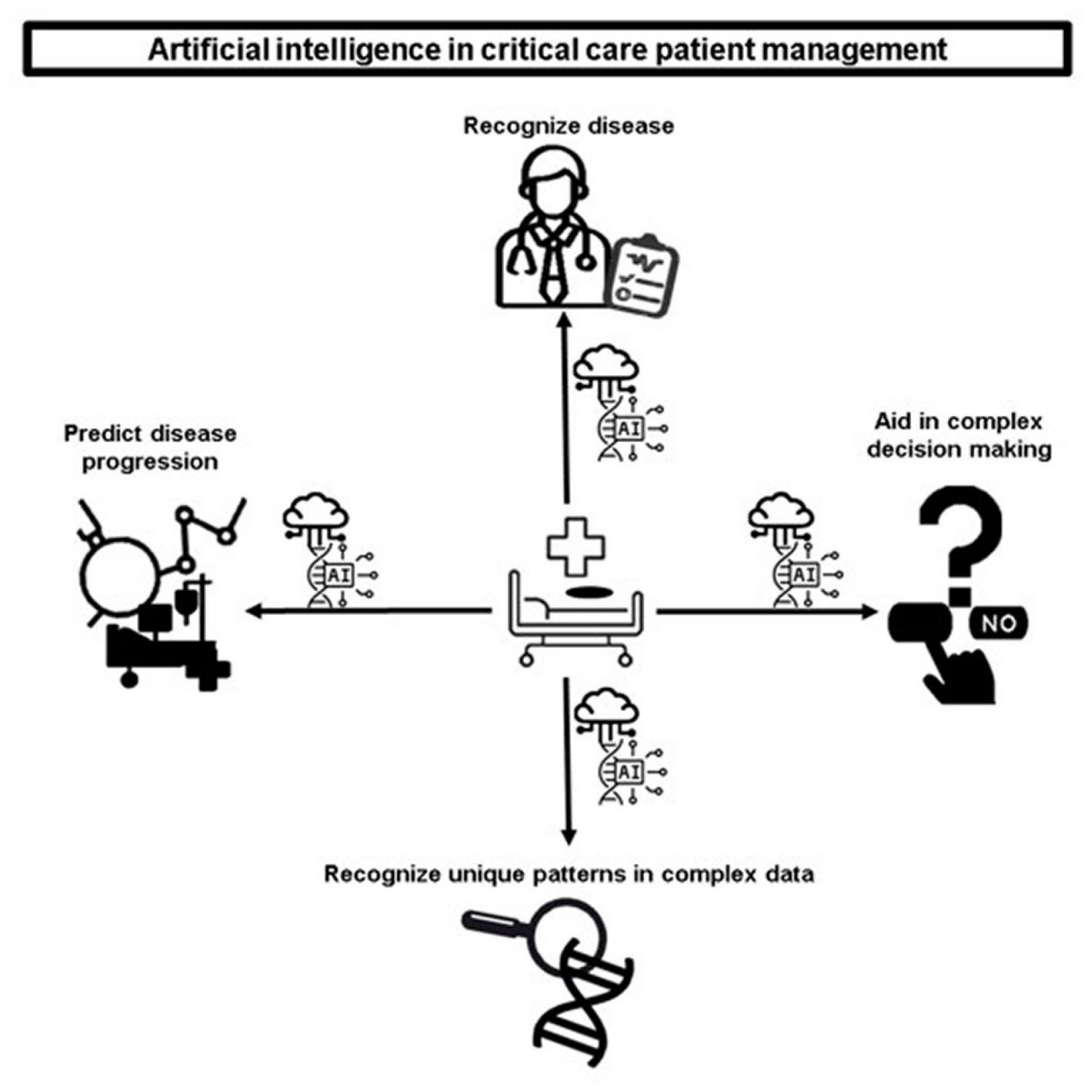
Artificial intelligence in critical care patient management.

AI has a multitude of diverse applications for the care of critically ill patients. The Figure 1 includes the recognition of disease, the prediction of disease progression, and the recognition of unique patterns in complex patient data. AI can also significantly aid caregivers in complex decision-making, as shown in Figure 1.

### 3.1. Recognition of disease

Diagnosing the source of a critically ill patient’s clinical decline can be a complex task due to the subtle onset of the disease or the presence of other conditions that obscure the main issue. Properly understanding the underlying context can be a challenging feat. For example, the presence of pulmonary infiltrates does not always indicate an excessive accumulation of fluid in the air sacs; it could be a sign of cardiac-related pulmonary edema, fluid in the pleural cavity, inflammation- or infection-related fluid buildup, or blood collections from trauma. Without proper clinical context and additional testing, appropriate and prompt treatment may be hindered. Artificial intelligence (AI) can aid in the medical diagnosis of critically ill patients by utilizing its advanced text and image processing abilities (4). A machine learning model, for instance, can differentiate congestive heart failure from other lung diseases and quantify pulmonary edema using a technique that provides a probabilistic manner for describing an observation (5). Furthermore, recent advancements in image analysis using convolutional neural networks have enabled the evaluation of traumatic brain injury with more accuracy than manual methods when viewed on head computed tomography scans (6). In a retrospective analysis by Prasad et al. (7), a reinforcement learning (RL) approach was used to develop a treatment protocol for electrolyte replacements in an ICU setting. This system provides recommendations for patient care that can be continuously updated based on the patient’s specific needs. The RL algorithm used available data from electronic health records, including vital signs, lab test results, and information about administered drugs and procedures, to estimate a patient-specific protocol for electrolyte repletion at six-hour intervals. The recommendations were presented by the AI algorithm in an interpretable and hierarchical manner, with the system first suggesting whether electrolyte replacement is needed and the best route for it, followed by the most appropriate dosage if the clinician chose to administer it. The RL system provided a more controlled and data-driven approach to electrolyte repletion as compared to traditional provider- or protocol-driven methods, which are often prone to error and deviation. This system also allows for greater flexibility and adaptability, considering patient context and clinical priorities. Optimal RL policy is reported to be able to recommend electrolyte replacements in a more targeted manner, potentially reducing the number of repletion events and the cost and time associated with unnecessary or repeat orders. Additionally, the system uses a reward and punishment system, reducing the costs and risks associated with intravenous delivery (7). This is not to underscore the value and significance of care-givers in the critical care setting; instead, it is a remarkable example of how new technologies such as AI can have a significant impact on the care of critically ill patients.

### 3.2. Prediction of disease progression using random forest models

Predicting disease progression is crucial for critically ill patients, as a delay in detecting clinical instability can result in harm or death (4). A dynamic random forest model is a type of machine learning algorithm that can be used to predict outcomes in the critical care setting. It works by using an ensemble of decision trees that can adapt and update in real-time as new data becomes available. A study by Yoon et al. (2) found that a dynamic model using random forest classification could predict cardiorespiratory instability, defined as a combination of hypotension, tachycardia, respiratory distress, or decreased oxygen saturation 90 min before it occurred in reality (2, 4). The use of AI and machine learning has expanded across various fields such as public health, disease prediction, and drug development, including the ability to predict viral mutations before they arise (4). The power of AI approaches continues to be utilized in a wide range of disease prediction and drug development applications (8). In a study by Davoudi et al. (9), tachycardia, which frequently precedes shock, was predicted 75 min before its onset using a random forest model (9). Although not in the critical care setting, hypotension was also predicted prior to its occurrence in the operating room and confirmed by a randomized controlled trial, reducing the rate of intraoperative hypotension to 1.2% (10, 11). In the critical care space, the prediction of hypotension events in the ICU has already been achieved using a random forest model that analyzed electronic health records and vital signs data, with 92.7% sensitivity, 15 min before the event even occurred (12). Another area where machine learning, a subset technology of artificial intelligence is in the assessment of pain in critically ill patients. In a study by Kobayashi et al. (13) which focused on using machine learning to assess the pain experienced by ICU patients, reported that vital signs, which are measured continuously in the ICU, can be used to predict pain with an accuracy upwards of 85% using a random forest (RF) model. This shows that machine learning can be used to continuously evaluate pain, which is important for pain management and the use of pain medication in ICUs. Their study also suggests that the use of an automated and continuous pain assessment algorithm may help relieve pain in patients who cannot communicate which could improve their life expectancy (13). All these examples show how the utilization of such models can prove significantly useful for management of critically ill patients.

### 3.3. Recognition of unique patterns in complex data

Critical illness is a complex condition that presents itself in various and unpredictable ways, leading to organ dysfunction and complicating the disease and recovery processes. To effectively manage these critical states, a careful consideration of underlying etiologies and clinical conditions is necessary. AI can help by recognizing unique patterns within complex data and identifying specific phenotypes or endotypes that reflect the individual’s critical state, leading to more personalized treatment plans (14). This relies heavily on access to large amounts of training data and phenotypic information. The complexity of medical care is highlighted by the fact that the same symptoms can be caused by different underlying conditions, making it difficult to provide personalized treatment. Diseases such as brain disorders, cardiovascular issues, and digestive problems are examples of this complexity. Innovative techniques and tools have been used to achieve personalized phenotyping in patients, combining practical experiences and scientific knowledge to realize the potential for using AI in a systems medicine approach to personalize medical care (15). The advancement of AI techniques has enabled researchers to uncover the underlying causes of various phenotypes, including genetic variations and cancer diseases, and by utilizing these tools and combining them with other methods, the biomedical field will be able to advance their knowledge and understanding of the relationship between genomics and expressions in diseases, promising faster and more accurate discoveries (16). This exemplifies how AI can serve as an aid to personalized patient care for critically ill patients.

### 3.4. Aid to complex decision-making in critical care

AI has the potential to assist doctors in the complex process of assessing patient risk levels for treatments, determining those who are most likely to experience a sudden deterioration, and analyzing multiple small outcomes to enhance overall patient outcomes. However, the complexity of AI techniques can affect physician comprehension and interpretation of results (17). To overcome this challenge, it is important for medical education to involve physicians in model creation and educate them in this field. AI platforms have the potential to be more efficient in some aspects compared to caregivers. For example, when compared to senior consultants, an AI platform such as Childhood Cataract Cruiser has proven to be more efficient and time-efficient for diagnoses, with high patient satisfaction rates (18). Such platforms can also be tested in the critical care setting. If they prove successful, it could significantly increase the efficiency of care delivery in the ICU. One-size-fits-all solutions are not effective in dealing with complex problems, as evidenced by the lack of improvement in septic shock outcomes in recent years despite various treatment guidelines (19, 20). Utilizing the concept of reinforcement learning has the potential to offer individualized solutions to the diverse nature of septic shock and varying host responses. A study by Komorowski et al. (21) used reinforcement learning on time series data with 44 features collected from mechanically ventilated patients, which resulted in improved outcomes compared to standard clinical care, reducing 90 day and ICU mortality rates (21). AI can also perform real-time electrocardiogram analysis to detect myocardial infarctions. A study by Chen et al. (22) reported using AI-assisted real-time analysis of electrocardiograms in the prehospital setting and found that it was feasible and had the potential to reduce delays in treatment times for patients requiring percutaneous coronary interventions (22). These examples demonstrate the use of AI for therapeutic guidance in medical decision-making for critically ill patients with good efficacy.

### 3.5. Intelligent decision making intervention in critical illness

By assisting in decision-making and enabling healthcare professionals to concentrate their efforts on investing more time with patients, artificial intelligence can help to promote shared decision-making (SDM) (22). AI technologies offer a wide range of information and have the capacity to evaluate enormous amounts of data and find correlations that scholars and healthcare professionals would have overlooked (23). The bioethics of employing AI for health decision-making, the challenges involved, patients’ and healthcare practitioners’ perspectives of AI-based decision aids, and how it should be included to provide patient-centered healthcare are all topics of developing study. Nevertheless, little is known about the actual application of AI in SDM or how it may help with the decision-making phase of SDM.

## 4. Challenges and obstacles to AI in critical care patient management

**Figure 2:**
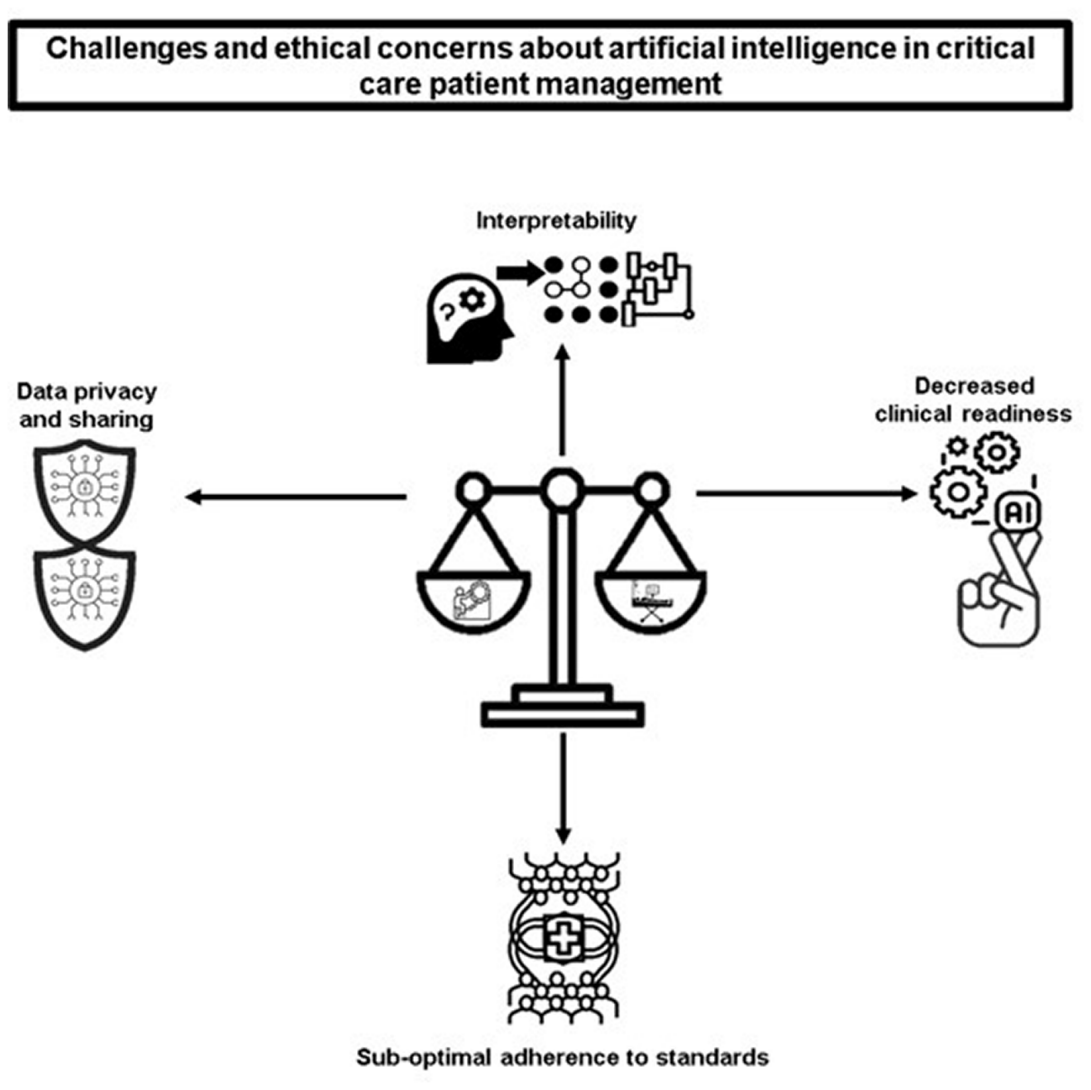
Challenges and ethical concerns of AI in critical care patient management.

Despite the potential benefits of AI in healthcare, particularly in the critical care setting, it is important to be aware of the potential challenges and obstacles that may arise when implementing AI models for critically ill patients. These roadblocks should not be ignored or overlooked, as they can have significant consequences for patient care and outcomes. The Figure 2 includes interpretability, data privacy and sharing, decreased clinical readiness and sub-optimal adherence to standard. A figure depicting the challenges and ethical concerns of AI in critical care patient management is shown in Figure 2.

### 4.1. Interpretability of AI in the intensive care unit

The deployment of AI in a healthcare setting, specifically at the bedside, requires careful planning and consideration of key factors such as usability and trustworthiness. The involvement of all relevant stakeholders, including patients, clinicians, researchers, and hospital administrators, is crucial for the success of the deployment. To ensure that the AI systems are effective and well-received, the implementation strategy should focus on creating models with a manageable amount of information that is presented in an understandable and visually appealing manner. This can be achieved through the use of interpretable logic and a user-friendly graphic interface. One of the key challenges in deploying AI systems at the bedside is ensuring that the AI-generated alerts are accurate and not overwhelming, so as to prevent alarm fatigue (23). In recent research on predicting hypotension in the ICU, the use of a stacked random forest model was found to reduce the number of alerts tenfold while still maintaining accuracy (12). To build trust and acceptance of AI systems among end users, it is important to understand the AI-generated predictions and recommendations. Despite the complex nature of many AI models, researchers are working to enhance their interpretability. The creation of a graphic user interface is essential for the effective deployment of AI systems at the bedside, as it helps to improve hospital workflow and reduce the burden on healthcare workers. Additionally, the use of deep learning in the analysis of patient behavior and environmental stimuli can provide useful information for detecting delirium in ICU patients (9). Care-takers are keen to understand how machines arrive at predictions that involve patient care. There are different software technologies that can help caregivers to understand how machines arrive at these predictions. One such example is the Shapley additive explanation (SHAP), a method that explains how machines arrive at individual predictions. In a study by Alderden et al. (24), the risk of developing hospital-acquired pressure-related injury (HAPrI) was analyzed in COVID-19 patients who were hospitalized in the ICU. The study aimed to utilize machine learning algorithms to create a predictive model for HAPrI risk and ensure that the model was transparent and understandable for medical professionals. The best-performing model was an ensemble SuperLearner, which showed good discrimination in HAPrI risk assessment. The use of explainable AI methods such as SHAP plots was a novel approach in this study and provided a way to visualize the relationships between the patient’s characteristics and the predictions made by the model. This study found that COVID-19 positive critical care patients have a higher risk of HAPrI compared to non-COVID patients. The use of machine learning algorithms to evaluate HAPrI risk in COVID-19 patients in the ICU is reported to be a feasible approach, and explainable AI methods such as SHAP plots provide a means of ensuring that the model is understandable and trustworthy for medical professionals. Medical professionals need to understand how the model reached its decisions for each individual patient to decide whether the model is trustworthy for that patient (24). Care-takers generally have a positive attitude towards the adoption of AI. Mlodzinski et al. (25) set out to examine the perspectives of both healthcare providers and non-providers regarding the use of machine learning (ML) in critical care. The study found that both groups generally have positive attitudes towards the use of ML in healthcare; however, non-providers with more knowledge about ML and AI are more likely to feel favorable towards its use. The study also found that there were no major differences in the level of comfort or knowledge among providers, regardless of their level of experience. Furthermore, the study identified common concerns such as systemic bias in data, patient safety, negative effects on the doctor-patient relationship, and data privacy an security. Among providers, workflow interruptions were also identified as a major concern, while limited knowledge of ML and AI was a concern among non-providers. It provided important insights into provider and non-provider perspectives on ML-based tools and will play a crucial role in optimizing their clinical utility (25). In the future, it will be important to design ICU systems that embrace the capabilities of AI and address caregiver concerns in order to enable early detection of patient deterioration and improve the accuracy and trustworthiness of AI-generated predictions. The complex nature of many AI models often makes it difficult to understand the rationale behind the computation and output, leading to resistance among healthcare professionals to adopting these models in daily practice. The fear of performing unnecessary interventions or changing treatment strategies without scientific evidence can have serious consequences, especially in critical care where patient outcomes are directly linked to such decisions (26, 27). However, there are efforts underway to address the issue of complex AI models. ML techniques are being used to determine what kinds of strategies caregivers use to make their decisions. For example, using game theory to measure the importance of features in predicting near-term hypoxic events during surgery has helped explain the contribution of various features to the AI model’s output. This approach has been shown to provide consistent results with prior knowledge and literature, leading to improved clinical decision-making and preventing hypoxia during surgery (28). This can also be extrapolated to the critical care setting to explain the contribution of different features in the output of AI models. Additionally, providing detailed methodologies for model validation, robustness of analysis, and expert knowledge can help alleviate concerns and increase the reliability and trust in AI models (4).

In this study, in contrast to SHAP, we will concentrate on two more example post-hoc model accuracy techniques that have gained minimal attention in the physical scientific world, namely breakDown (BD) research and Ceteris-Paribus (CP) analyses. The BD technique, like the SHAP method, is founded on the variety attribution principle, which divides each observation’s estimate into its individual variable components (29, 30). The BD values offer action descriptions of the impacts of variables in a clever way, in contrast to the SHAP values. The independence and non-interaction of the input characteristics (factors or descriptors) constitutes a component of the BD method’s presumptions (31). For BD evaluation, there are two algorithms: step-up and step-down. The step-down approach begins with a complete collection of input characteristics.

Finally, in order to minimize the proximity to the prediction models, each selected feature contribution is determined by successively eliminating one characteristic from a set accompanied by variable relaxation. In contrast to the step-down approach, the step-up method begins with a null set and proceeds in the other manner. In feature contributions, both techniques have been proved to deliver consistent results.

On the contrary hand, the CP profiles, also known as individual conditional expectancies (ICE) plots, assess the impact of a variable from a learned ML model while assuming that the levels of all other variables remain constant (akin to what-if analysis).

Using CP profiles, one can quickly see how the source and responses are connected and how the projected response depends on a characteristic (e.g., in a non-linear, linear or complex). In this approach, the CP analysis aids in quantifying the influence of a particular variable on the conclusions drawn from a black version and offers a brief, visual description of the functional form linking an input with an output. From either the SHAP or BD analysis, it is difficult to draw conclusions regarding this type of functional reliance. Hence, adding CP profile plots to SHAP and BD studies is of great utility.

### 4.2. Reproducibility issues of AI systems during application

Frequently, determining the causative factors of deteriorating patients from the complete list of differential diagnoses is tough, because of the subtle feature of early illness progression or the existence of co-existing disorders disguising the underlying problem. Above all, it is important to accurately interpret the underlying context, which is sometimes difficult to do. For instance, it is not enough to infer that pulmonary infiltrates are caused by an excessive amount of alveolar fluid. These may signify pleural effusion, pulmonary embolism fluid from an infection or inflammation, pulmonary edema with a cardiac origin, collections of blood due to trauma, or any of these conditions. Lacking clinical context and additional testing, proper and prompt care might be delayed. AI might aid in such circumstances by obtaining a more exact diagnosis, given enhanced text and picture processing power. Using a machine learning algorithm, congestive heart failure (CHF) could be distinguished from other cause of lung illness (32), and the quantity of pulmonary edema brought on by the CHF could be measured using semi-supervised machine learning and a finite difference autoencoder. An AI model was used to evaluate imaging data from hospitalized patients in during acute pulmonary syndrome coronavirus 2 (SARS-CoV-2) epidemic in order to identify coronavirus disease 2019 (COVID-19) (33).

The application of AI in the clinical setting is hindered by a lack of sufficient clinical trials and experiments, leading to a low rate of reproducibility and future analysis. A review of 172 AI solutions created from chart data revealed that the clinical readiness level of AI was low, with 93% of the analyzed solutions falling below stage 4 for real-world application and only 2% undergoing prospective validation (29). The reproducibility of AI solutions is uncertain due to limitations in data openness and algorithmic complexity, and there are no clear protocols in place to examine this thoroughly. A study showed that attempting to reproduce mortality prediction projects resulted in large sample size differences in half of the experiments, highlighting the importance of accurate labeling, clinical context, and precise reporting methods (30). Adherence to reporting standards and the risk of bias are also sub-optimal, as a study of 81 non-randomized and 10 randomized trials using deep learning showed that only 6 of the 81 non-randomized studies had been tested in a real-world clinical setting, and 72% of the studies had high risks of bias (31). Even more complex AI models, such as reinforcement learning, face challenges as they require significant computational resources and are difficult to test on patients in a clinical environment. However, new approaches such as inverse reinforcement learning may offer a solution by inferring information about rewards, potentially making decision-assisting engines more robust and reliable with varying input data, which is crucial in critical care data science where data is vast and extremely diverse (34).

## 5. Ethical concerns

The use of AI in critical care is a new and developing field, and the ethical issues that may arise from its use are not fully understood. However, there are a few aspects that can be discussed to anticipate potential ethical dilemmas. One issue is data privacy and sharing. The process of collecting and manipulating data to find patterns could lead to the leakage of confidential information, particularly during the pre-processing stage and external validation. De-identification and novel models such as federated learning might help to minimize data leakage and increase the speed of the validation process (4). Another ethical concern is the safety of AI models in patient care. The maturity metric used for self-driving cars has been used to describe the safety of AI models, with 6 levels ranging from no automation to full automation (35). Based on this scale, most AI-driven solutions would currently fall into the categories of partial or no automation, meaning that human oversight and decision-making are still required. This also raises questions about patient autonomy and informed consent, as AI recommendations may not always align with a patient’s preferences. In order to address these ethical issues and overcome the limitations of AI, researchers and clinicians need to be aware of the potential problems and develop solutions to mitigate them. This also includes understanding patient perspectives and incorporating them into the development of practical and ethical AI solutions (4).

## 6. Guidance for future

**Figure 3:**
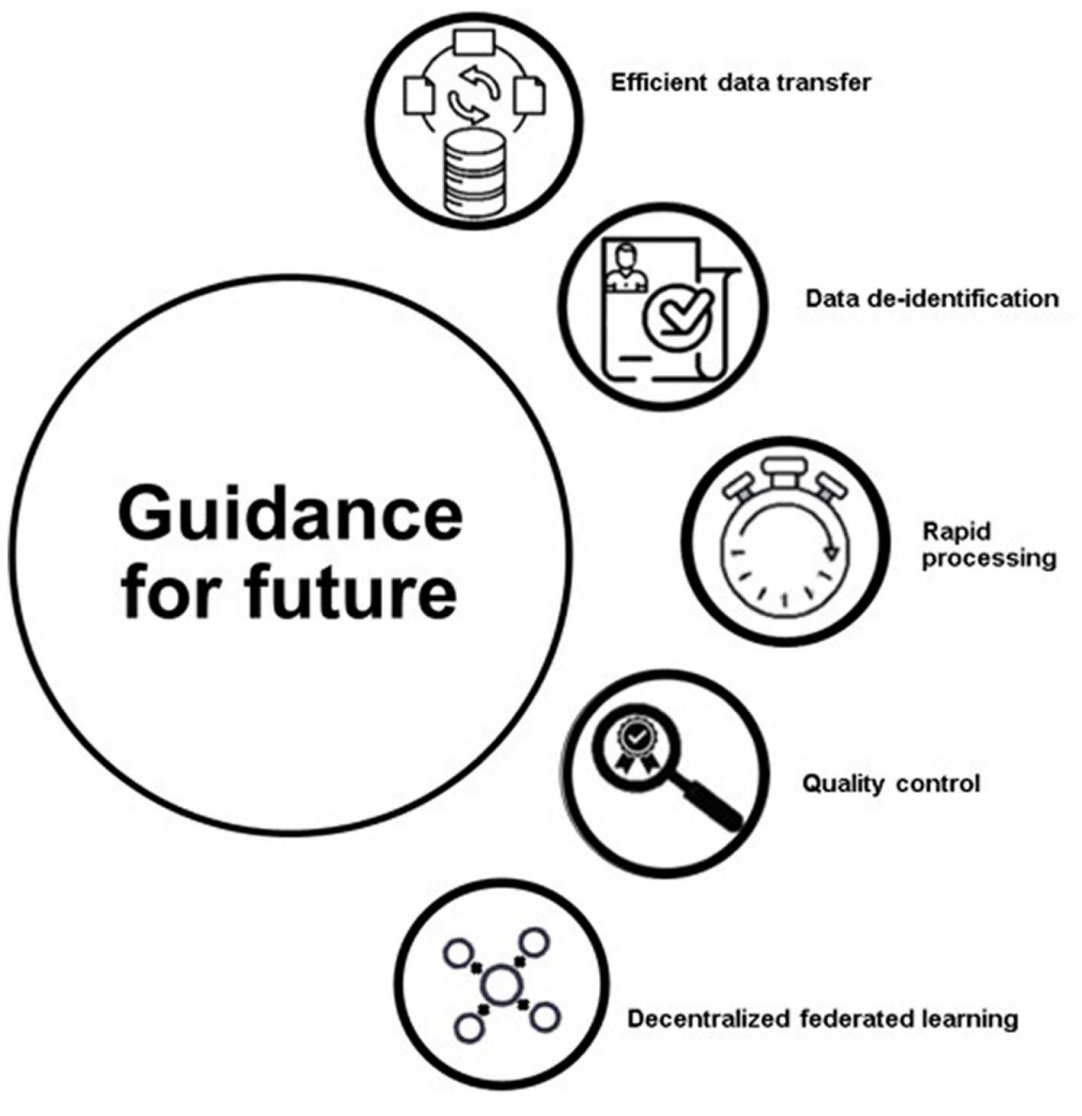
Guidance for future.

The Figure 3 includes efficient data transfer, data de-identification, rapid processing, quality control, and decentralized federated learning. The field of AI has the potential to greatly impact critical care, but there are several steps that must be taken in order to make this happen, as highlighted in Figure 3. Many of the recommendations have either already been implemented or are in the process of being implemented.

One of the most important is ensuring that the data used for training AI models is properly de-identified and standardized. This is important for both privacy and data quality, as data from different hospitals may be structured differently and contain different amounts of personal information. The Society of Critical Care Medicine and the European Society of Intensive Care Medicine have developed a process for de-identifying data, that involves separating personal data from anonymous data, conducting a risk-based process to de-identify the personal data, and conducting an external review to ensure that all privacy and legal considerations are met (36). Another important step is standardizing the data in order to facilitate efficient exchange between different hospitals. This requires developing a standard format for storing and exchanging clinical and physiological data. One such format, the Hierarchical Data Format, Version 5 (HDF5), allows for the storage, compression, and real-time streaming of multiparameter data. This would allow for the integration of other types of large-scale datasets, such as those in imaging or genomics (37). Another solution is the use of federated learning, where models can be trained locally at different hospitals rather than having the data sent to a central location for training. This helps to preserve privacy and can be particularly useful when the data distribution is imbalanced or skewed. A successful example of this approach was seen during the COVID-19 pandemic, where 20 academic centers collaborated to predict clinical outcomes from COVID-19 using a federated learning approach. The AI model was trained on chest X-ray data, and achieved an average area under the curve (AUC), of 0.92 for predicting 24–72 h outcomes (4). The task of labeling events for AI models can be labor-intensive and resource-intensive, but novel AI models, such as weakly supervised learning, are being developed to make the process more efficient. This type of learning can build desired labels with only partial participation of domain experts, which preserves resources. Additionally, clinical trials can also be designed with AI models to maximize benefits and minimize risks to participants, as well as to make the best use of limited resources. One example of this is the Randomized Embedded Multifactorial Adaptive Platform for Community-Acquired Pneumonia (REMAP-CAP) trial, which uses a Bayesian inference model, to identify the optimal treatment for community-acquired pneumonia and has contributed to improved survival among critically ill COVID-19 patients (38). The labeling process for AI models can be a difficult and resource-intensive task. To make this process more efficient, new AI models such as weakly supervised learning have been developed. This method of learning allows for the partial involvement of domain experts and can reduce resource usage. For example, in the case of COVID-19 patients visiting the emergency department, weakly supervised learning was used in conjunction with medical ontologies and expert-driven rules to classify patients with related symptoms. This combination of weakly supervised learning and pretrained language models improved performance compared to a majority vote classifier, reducing the cost of creating classifiers in a short period of time, especially during a pandemic when experts may not be available for labeling. Innovative trial designs can also be developed with AI models to make the best use of resources and minimize risks to participants (39). This platform, initially developed for community-acquired pneumonia, has continued to enroll patients during the COVID-19 pandemic and has contributed to improved survival among critically ill patients (4, 40–42). For an AI model to be useful in real-life settings, it needs to provide important information in a timely manner, especially for critically ill patients who require quick feedback. The AI model should have a fast data pre-processing platform, parsimoniously feature input data, and deliver output rapidly. To date, no such model has been developed that can successfully do the above-mentioned tasks in such a quick manner. Although true real-time prediction is a challenging task, the application of a real-time AI model in the critical care environment could offer significant benefits without delay. Once the AI model is deemed useful in a clinical setting, quality assessment efforts should follow to ensure its maturity and integration with healthcare. The National Academy of Medicine of the United States has published a white paper on AI use in healthcare, emphasizing the development of guidelines and legal terms for safer, more effective, and personalized medicine (43).

## 7. Conclusion

The utilization of artificial intelligence (AI) in critical care presents numerous opportunities for enhancing outcomes in critically ill patients by enabling the perception of disease, predicting changes in pathological processes, recognizing unique patterns in disease presentations, and assisting in the process of clinical decision-making in a symbiotic fashion with care-givers. Moreover, AI can facilitate the understanding of medical processes by presenting recommendations for patient care in an interpretable and hierarchical manner through techniques such as reinforcement learning. The technology has the potential to improve understanding of the diverse clinical needs of critically ill patients, risk assessment for treatments, and the analysis of patient outcomes.

## Author contributions

MS and MI: data curation, formal analysis, investigation, conceptualization, supervision, visualization, writing – original draft, and writing – review and editing. HM: data curation, formal analysis, investigation, methodology, writing – original draft, writing – review and editing, and supervision. All authors reviewed the final version of the paper and approved it for submission and publication.

## Conflict of interest

The authors declare that the research was conducted in the absence of any commercial or financial relationships that could be construed as a potential conflict of interest.

## Publisher’s note

All claims expressed in this article are solely those of the authors and do not necessarily represent those of their affiliated organizations, or those of the publisher, the editors and the reviewers. Any product that may be evaluated in this article, or claim that may be made by its manufacturer, is not guaranteed or endorsed by the publisher.

## Abbreviations

AI: Artificial intelligence
ML: Machine learning
RL: Reinforcement learning
ICU: Intensive care unit
CCM: Critical care medicine
RF: Random forest
SHAP: Shapley additive explanations
HAPrI: Hospital-acquired pressure-related injury
COVID-19: Coronavirus disease – 2019
HDF5: Hierarchical data format-version 5
AUC: Area under the curve
REMAP-CAP: Randomized embedded multifactorial adaptive platform for community-acquired pneumonia
